# Reducing Health Inequalities in Individuals with Severe Mental Disorders: Harnessing Real-World Data and Patient-Reported Measures

**DOI:** 10.3390/jcm12134481

**Published:** 2023-07-04

**Authors:** Laurent Boyer, Pascal Auquier, Dong Keon Yon, Pierre-Michel Llorca, Guillaume Fond

**Affiliations:** 1 CEReSS-Health Service Research and Quality of Life Center, Aix-Marseille University, 13005 Marseille, France; pascal.auquier@univ-amu.fr (P.A.); guillaume.fond@gmail.com (G.F.); 2 Center for Digital Health, Medical Science Research Institute, Kyung Hee University College of Medicine, Seoul 130-701, Republic of Korea; yonkkang@gmail.com; 3 Department of Pediatrics, Kyung Hee University Medical Center, Kyung Hee University College of Medicine, Seoul 130-701, Republic of Korea; 4 Department of Psychiatry B, CHU Clermont-Ferrand, Institut Pascal, Axe TGI, CNRS-UMR 6602, Université Clermont Auvergne, 63011 Clermont-Ferrand, France; pmllorca@chu-clermontferrand.fr

Mental disorders are the leading cause of diminished lifespans worldwide and make up 5 of the top 10 most significant causes of disability [1,2]. In European Union member states, an estimated 80 million women and men of all ages currently experience some form of mental disorder. The combined financial burden of treating mental disorders is currently EUR 600 billion across all 27 EU members [3]. On average, people with severe mental disorders (SMD), such as schizophrenia, bipolar disorder and major depressive disorder, die up to 15 years earlier than unaffected individuals [4]. According to the Institute of Medicine, legal standards for treating patients with SMD are often insufficient, leading to non-patient-cnetric, delayed, inefficient, inequitable, and, at times, unsafe care [5]. Individuals with SMD have complex care needs that require significant financial resources [6]. It is vital that healthcare providers redesign care to improve patient outcomes.

To address this problem, recent research studies suggest that the appropriate and timely use of medication, such as second-generation long-acting injectables and clozapine in schizophrenia, when combined with psychological care, can reduce the mortality gap between individuals with and without mental disorders [4]. However, this primary care-centric approach is not sufficient to equalize treatment outcomes. It is also crucial to embrace precision psychiatry, explore the diagnostics potential of biomarkers, and investigate novel pharmacological therapies [7]. Recently, in France, the PROgram-project in Precision PSYchiatry (PROPSY) allocated EUR 80 million to the advancement of precision psychiatry. This scheme aims to enable tailored treatment options that reflect individual patients’ unique characteristics and care needs, leading to more effective and personalized care. However, a truly patient-centered approach to mental health care must integrate patient-reported outcome measures (PROMs), patient-reported experience measures (PREMs), and real-world data and evidence (RWD/RWE). Despite general recognition of their importance among experts, the actual use of these data and measures is insufficient. This editorial highlights the significance of PROMs, PREMs, and RWD/RWE to mental health care, explaining why utilizing them could enable patient-centered care and equalize care outcomes.

**How can we transition from measuring efficacy to measuring effectiveness?**

Randomized controlled trials and more modern designs, such as adaptive trials [8], experience problems related to external validity. Only 20% of individuals with schizophrenia take part in randomized clinical trials [9]. Therefore, trial results are based on the efficacy of care measured under experimental conditions that are not related to patients’ experiences. This problem presents a serious question: how can we transition from prioritizing experimental efficacy to prioritizing “efficacy in real-life”, which is also called effectiveness? Firstly, we need to define a measurement of effectiveness. Numerous past studies emphasized that assessing the effectiveness of psychiatric interventions must not only consider symptoms, as symptoms merely represent a fraction of an individual’s overall experience. Care-quality measures must also explore other dimensions, including functioning [10], quality of life [11], and recovery [12]. Symptom remission is not a prerequisite for functional remission and good quality of life [13]. Secondly, even if we can measure functioning, quality of life, and recovery, we currently only measure it while operating under experimental conditions. Our challenge, therefore, is to integrate PROMs into clinical practice [14].

**Why does effectiveness depend on the care experiences of patients?**

Another aspect to consider is the fact that the effectiveness of care is intricately linked to the patient’s experience of accessing the healthcare system, including their relationships with physicians and other healthcare professionals. The patient’s overall care experience has an impact on the effectiveness of treatment, primarily in terms of its relationship with adherence to treatment, particularly in individuals with chronic illnesses [15,16]. 

Moreover, two theoretical models can shed light on the additional non-pharmacological and biopsychosocial effects of a patient’s care experience [17]. The first model is the placebo response [18], which suggests that the interaction between patients and clinicians influences the likelihood that individuals experience placebo effects [19]. Positive relationships between patients and physicians that are characterized by open communication have been shown to alleviate symptoms [18]. Additionally, patients who perceive their clinicians as being empathetic also exhibit reduced levels of inflammation [20]. Socially transmitted placebo effects have also been reported, showing that care providers’ expectation that treatment will succeed can impact its efficacy [21]. The second model, which is known as the set and setting theory [22], further emphasizes the influence of non-biological factors on responses to treatment. This concept is crucial not only for pharmacological research [23], but also for advancing drug research and developing effective drug policies [22]. In the context of pharmaceuticals, the terms ‘set’ and ‘setting’ refer to the psychological and environmental contexts, respectively, that are known to shape responses to different medications. These theoretical models demonstrate that a patient’s experience of care extends beyond the traditional biomedical framework, emphasizing the importance of the therapeutic relationship, communication, empathy, and the broader context in which treatment is provided. Incorporating these factors into the delivery of care can profoundly impact treatment’s effectiveness. To this end, we developed seven PREMs that involved interviews with SMD patients and covered the following topics: relationships between patients and clinicians, respect and dignity, access and care co-ordination, drug therapy, provision of information, psychological care, and the care environment [24,25,26,27,28]. These questionnaires were made available via the Internet and adapted to patients’ response. Therefore, based on the principles of item response theory, only the most relevant items would be presented to patients, taking into account their initial or prior responses. This adaptive approach ensured that patients answered the most relevant questions, thus leading to more accurate score estimates. Furthermore, this method reduced the burden on patients relative to traditional fixed-format questionnaires. The main challenge lay in the implementation of PREMs in clinical practice by using digital platforms.

**How can we use “real-world databases” and health services-related research to better manage healthcare disparities?**

As the U.S. Food and Drug Administration and the European Medicine Agency both recognize synthetic control groups based on real-world databases as powerful sources of evidence that could replace randomized controlled trials, analyzing real-world data from cohort or national health care databases could provide insights for experts to leverage to better understand and address health-related disparities. These databases offer comprehensive insights regarding healthcare utilization, organization, and outcomes for individuals with SMD. By utilizing these data, past researchers identified significant disparities in outcomes from perinatal [29], COVID-19 [30,31,32,33,34,35,36,37,38], and cancer care [39,40]. These findings highlight the need to prioritize research into health disparities and tailor healthcare to suit the specific needs of disadvantaged populations. Improving care access and quality must remain a critical priority regarding treatment for individuals with mental disorders.

**Why is this roadmap important to the future of psychiatry?**

By following this roadmap, we can greatly reduce health disparities, improve mental healthcare outcomes, and promote equitable access to care for individuals with SMD.

**How can experts develop personalized psychiatric treatment strategies?**

By integrating PROMs and PREMs into clinical practice, healthcare providers can gain valuable insights into patients’ actual care experiences. This information would, thus, enables providers to customize care by focusing on functioning, quality of life, and recovery, rather than simply treating the symptoms of SMD.

Leveraging real-world databases and conducting health services-related research [41] allows for the systematic identification and addressing of healthcare disparities. This knowledge is vital to developing effective interventions and ensuring equitable access to high-quality care for all individuals with SMD.
